# Exploring the Immunoprotective Potential of a Nanocarrier Immersion Vaccine Encoding Sip against *Streptococcus* Infection in Tilapia (*Oreochromis niloticus*)

**DOI:** 10.3390/vaccines11071262

**Published:** 2023-07-20

**Authors:** Ye Cao, Jia Liu, Gaoyang Liu, Hui Du, Tianqiang Liu, Gaoxue Wang, Qing Wang, Ya Zhou, Erlong Wang

**Affiliations:** 1 Key Laboratory of Fishery Drug Development, Ministry of Agriculture and Rural Affairs, Key Laboratory of Aquatic Animal Immune Technology, Guangdong Province, Pearl River Fisheries Research Institute, Chinese Academy of Fishery Sciences, Guangzhou 510380, China; 2 Northwest A&F University Shenzhen Research Institute, Shenzhen 518000, China; 3 College of Animal Science and Technology, Northwest A&F University, Yangling 712100, China; 4 College of Animal Science and Technology, Chongqing Three Gorges Vocational College, Chongqing 404155, China; asian_chou@outlook.com

**Keywords:** *Streptococcus*, surface immunogenic protein, immersion immunization, Tilapia

## Abstract

Tilapia, as one of the fish widely cultured around the world, is suffering severe impact from the *streptococcus* disease with the deterioration of the breeding environment and the increasing of breeding density, which brings serious economic loss to tilapia farming. In this study, the surface immunogenic protein (Sip) of *Streptococcus agalactiae* (*S. agalactiae*) was selected as the potential candidate antigen and connected with bacterial nano cellulose (BNC) to construct the nanocarrier subunit vaccine (BNC-rSip), and the immersion immune effects against *S. agalactiae* and *Streptococcus iniae* (*S. iniae*) in Nile tilapia were evaluated on the basis of the serum antibody level, non-specific enzyme activity, the immune-related gene expression and relative percent survival (RPS). The results indicated that Sip possessed the expected immunogenicity according to the immunoinformatic analysis. Compared with the rSip group, BNC-rSip significantly induced serum antibody production and improved the innate immunity level of tilapia. After challenge, the RPS of BNC-rSip groups were 78.95% (*S. agalactiae*) and 67.86% (*S. iniae*), which were both higher than those of rSip groups,31.58% (*S. agalactiae*) and 35.71% (*S. iniae*), respectively. Our study indicated that BNC-rSip can induce protective immunity for tilapia through immersion immunization and may be an ideal candidate vaccine for controlling tilapia streptococcal disease.

## 1. Introduction

China is the largest tilapia breeding producer and exporting trade country. The tilapia industry has very important significance to China and even the world food supply and promoting the international trade of China. However, in recent years, tilapia disease occurs frequently, resulting in enormous economic losses for the tilapia industry. Disease occurrence has seriously obstructed the healthy development of the tilapia industry. *Streptococcus disease* is a bacterial disease that occurs frequently in a variety of freshwater and marine fish and is mainly caused by *Streptococcus agalactiae* (*S. agalactiae*) and *Streptococcus iniae* (*S. iniae*) [1]. *Streptococcus* infections have been reported daily worldwide in Tilapia (*Oreochromis niloticus*) [2]. *Streptococcus* can destroy the immune system and damage the organs, mainly causing fish septicemia and meningoencephalitis, and the diseased fish presented with typical clinical symptoms of streptococcicosis, including corneal opacity, exophthalmia, abnormal swimming and tissues hyperemia [3,4]. Group B streptococcus (GBS) has been a significant fish pathogen in recent years and is severely harmful to the fish industry, causing both morbidity and mortality [5,6]. The surface proteins located on the surface of bacteria are the first to be recognized by the host, triggering an immune response. Therefore, the ubiquitous surface proteins are ideal antigens for genetic engineering vaccines [5,7]. The surface immunogenic protein (Sip), as a kind of conserved protein in all GBS serotypes, has been demonstrated as a promising candidate in the development of *Streptococcus* vaccines [8]. A previous study has shown that *Sip* is highly conserved among different *S. agalactiae* strains like serotype Ia/c, Ib, II/R, III, V and VI, with 98% identity in the nucleotide sequences [9]. In addition, some studies have reported that Sip is an effective vaccine candidate antigen against *S. agalactiae* infection in tilapia [10].

For fish disease prevention and control, antibiotics are the main means of drug treatment at present, but because of the potential environmental pollution and food safety problems, this treatment is widely criticized. Vaccination has served as a broadly accepted and effective approach to the prevention of most pathogenic diseases in aquaculture [11]. Vaccines are mainly divided into inactivated vaccines, live attenuated vaccines, subunit vaccines and DNA vaccines. The safety and efficacy of live attenuated vaccines are questionable; the maintenance time of inactivated vaccines is limited, while DNA vaccines are mainly injected for immunity and are not suitable for large-scale farming [12]. However, subunit vaccines have higher safety and stability and are suitable for large-scale production. Immunization routes of fish vaccines mainly include injection, oral, and immersion immunization. Oral immunization is immunologically inefficient and easily decomposable, while injection immunization is time consuming and laborious [13]. As for immersion immunization, which is an ideal method of fish vaccination, allows fish to be inoculated in large-scale with little labor and damage to the fish, but the immune response is not strong enough compared with that of injection immunization due to the less of effective antigen entering the body. The nanocarrier strategy is one of the effective measures to figure out these issues. Bacterial nanocellulose (BNC), which has outstanding properties such as excellent permeability, stiffness, low density, biocompatibility, and renewability, is beneficial to the design of novel drug, protein and vaccine as the carrier delivery systems [14,15].

In this study, Sip was selected as the candidate antigen to prepare the subunit vaccine and linked with functionalized BNC to construct the nanocarrier vaccine system BNC-rSip. Based on the high consistency of Sip between *S. agalactiae* and *S. iniae*, the cross-protection effects of BNC-rSip against these two pathogens infections were evaluated in tilapia after immersion immunization.

## 2. Materials and Methods

### 2.1. Fish and Strain

Healthy tilapia (10.50 ± 0.50 g) was purchased from a fish farm in Guangzhou (Guangdong Province, China). The water temperature was 26–28 °C, the dissolved oxygen was kept above 6 mg/L, and the pH value was between 7.5 and 8.5. Tilapia was fed three times daily, and the residual feed was cleaned regularly and the water was changed on schedule. The experiment was carried out after 3 weeks of temporary rearing. *S. agalactiae* (serotype Ia) and *S. iniae* were cultured, identified, and stored in our laboratory. Prior to use, *S. agalactiae* and *S. iniae* were cultured in Brain–Heart Infusion broth (BHI) for 24 h at 37 °C, then centrifuged to discard the medium and adjusted to the needed concentration using Phosphate-Buffered Saline (PBS) buffer. The entire experimental procedure was approved by the Institutional Review Board of the Animal Experimental Ethics Committee, Northwest A&F University.

### 2.2. Amplification and Immunoinformatic Analysis of Sip

The genomic DNA was extracted from *S. agalactiae* using the Bacterial Genomic DNA Extraction Kit (Accurate biotechnology, Hunan, China). The specific primers (Sip-F: 5′-CATGCCATGGGCATGGAAACAGATACGACGTG-3′; Sip-R: 5′-CCGCTCGAGTTATTGTTAAATGATACGTG-3′) were designed based on the published Sip sequence of *S. agalactiae* in NCBI website (Genbank Number: AOV93705.1). The *Sip* amplification was achieved by PCR with the extracted genomic DNA and specific primers, followed by ligation with the pMD19-T vector (Takara, Beijing, China) to construct the pMD19-T-Sip plasmid. The identification of pMD19-T-Sip plasmid was conducted via PCR, followed by double enzyme digestion and sequencing (Tsingke biotechnology, Beijing, China). The obtained nucleotide sequence was submitted to NCBI GenBank to obtain the accession number. The conserved domains were analyzed using Conserved Domain Database (CDD) in NCBI [16].The conserved motifs of Sip in this study and the other 18 reference bacteria were checked via the MEME software (Version 5.5.3) [17]. The vaccine immunogenicity and immune response patterns were simulated using the C-ImmSim software [18], which predicted the candidate vaccine model antigen epitopes based on the principle of Position Specific Scoring Matrix (PSSM) and used the software to predict immune response interactions based on the principle of classical immunology. The C-ImmSim can detect the ability of vaccines to stimulate immune cells and molecules, such as B lymphocytes, helper T cells (Th cells), cytotoxic lymphocyte cells (CTL cells), natural killer cells (NK cells), dendritic cells (DC), immunoglobulins, and cytokines, and describe the cellular and humoral immunity induced by the vaccine.

### 2.3. Protein Expression and Immunogenicity Analysis of rSip

After double enzyme digestion of pMD19-T-Sip, the target band Sip was recycled using an Agarose Gel DNA Purification Kit (Accurate biotechnology, Hunan, China) and ligated with a double enzyme-digested pET28a vector to construct the recombinant expression plasmid pET28a-Sip. Then, it was transformed into *E. coil* BL21 (DE3) competent cell (Tsingke biotechnology) to obtain the expression bacteria BL21(pET28a-Sip). The recombinant protein rSip was induced expression with Isopropyl β-D- Thiogalactopyranoside (IPTG). The band size was verified via 12% SDS-PAGE. The immunogenicity of rSip was confirmed by Western Blotting with rabbit anti-*S. agalactiae* and anti-*S. iniae* serums (prepared by our lab) as the primary antibodies, respectively.

### 2.4. Preparation of BNC-rSip Vaccine and Vaccination

To construct the nanocarrier vaccine system BNC-rSip, dehydration condensation reaction was carried out to conjugate the BNC with Sip protein by linking the carboxyl groups in BNC and the amino groups in Sip protein [19]. After two weeks of temporary rearing, tilapia was randomly divided into four groups. Each group contained two tanks and immersion immunized with PBS, BNC, rSip vaccine, and BNC-rSip nanovaccine (dispersed in PBS buffer), respectively. Table 1 showed the groups, vaccine concentration, and immersion times. During the vaccination, 70 fish in each group (35 fish/tank) were immersed in 4 L water where the vaccine was homogeneously dispersed. After immersion vaccination for 8 h with continuous oxygenation, the vaccinated fish were placed back into the rearing tank for daily feeding.

### 2.5. ELISA Analysis of Serum Specific Antibody Level

The fish serum was collected from 1 to 4 weeks post immunization (wpi) to detect the specific antibody level using ELISA (enzyme-linked immunosorbent assay). Briefly, the collected serum was diluted in a ratio of 1:200 with coating buffer, added to 96-well plates (3 replicates per sample), and incubated for 12 h at 4 °C. Then, 5% skim milk powder was added, followed by incubation for 2 h at 37 °C. After incubation, 100 μL diluted rSip (diluted to 2 μg/mL) was added and incubated for 1 h at 37 °C. The mouse anti-6×His tag monoclonal antibody (BBI, Shanghai, China) and HRP-conjugated goat anti-mouse IgG antibody (BBI, Shanghai, China) were selected as the primary and secondary antibodies and were diluted 1:2000, respectively. Each step was washed three times (3 min each) with PBST (Phosphate-Buffered Saline with Tween-20). After that, 100 μL 3,3′,5,5′-Tetramethylbenzidine (TMB) chromogenic solution (Solarbio, Beijing, China) was added to each well and placed at 37 °C for 3–5 min in the dark. To terminate the reaction, 50 μL 2 M H_2_SO_4_ was added and the experimental results were measured within 10 min using a microplate reader (Molecular Devices Corp., Palo Alto, CA, USA) at 450 nm.

### 2.6. Determination of Serum Nonspecific Enzyme Activity

To analyze the nonspecific immune response after immunization, the serum enzyme activities were determined from 1 to 4 wpi, which included lysozyme (LZM), acid phosphatase (ACP), and alkaline phosphatase (ALP) activities. The detection methods were carried out according to the corresponding enzyme activity test kits (Nanjing Jiancheng, Jiangsu, China).

### 2.7. Evaluation of Immune-Related Genes Expression by RT-qPCR

The spleen tissues were sampled to determine the expression levels of immune-related genes at 3 wpi. Total RNA was extracted using the Trizol method, the concentration and purity of the extracted RNA were determined by spectrophotometer, and the RNA was reverse transcribed into cDNA using the commercial kit (Yugong Biology, Jiangsu, China). The expression levels of immune-related genes, including IgM, IL1β, IL-6, TNF-α, IFN-γ, CD4-1, CD8α, MHC I, and MHC II, were measured by RT-qPCR using a CFX96 real-time quantitative PCR system (Bio-Rad, CA, USA) with the following condition: 95 °C for 5 min; followed by 40 cycles at 95 °C for 20 s, and 60 °C for 1 min. Primers were designed and synthesized based on the sequence of each gene (shown in Table 2). EF1α served as the internal reference gene. The assay data were analyzed via the 2-ΔΔCt method [20], and three replicates were set for the assay.

### 2.8. Challenge and Protective Effect Evaluation

Sixty fish per group were randomly selected and divided into two subgroups (thirty fish/ subgroup) for *S. agalactiae* and *S. iniae* challenge tests at 4 wpi. Briefly, *S. agalactiae* and *S. iniae* were cultured in BHI medium for 24 h and adjusted to the needed concentration (5 × 10^7^ cfu/mL of *S. agalactiae* and 5 × 10^8^ cfu/mL of *S. iniae*, respectively) with PBS. The tilapia was intraperitoneally injected with 100 μL *S. agalactiae* and/or *S. iniae* suspension. The health status and mortality of challenged tilapia was monitored for 14 days. The dead fish were dissected and detected for bacterial infection in time. The relative percent survival (RPS) was computed in terms of the formula: RPS = [1—mortality (vaccinated group)/mortality (control group)] × 100%.

### 2.9. Statistical Analysis

All data were presented as the mean ± standard deviation (SD). Analysis of the data was conducted using the one-way ANOVA method. All statistical analyses were accomplished using GraphPad Prism version 9.0 (GraphPad Prism Software, CA, USA). In all cases, the significant differences were considered as *p* < 0.05.

## 3. Results

### 3.1. Immunoinformatic Analysis of Sip

**Figure 1:**
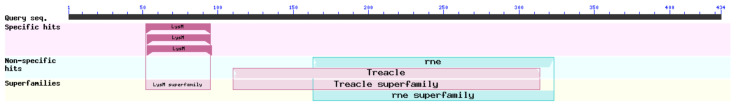
Conserved domain analysis of vaccine candidate antigen Sip.

**Figure 2:**
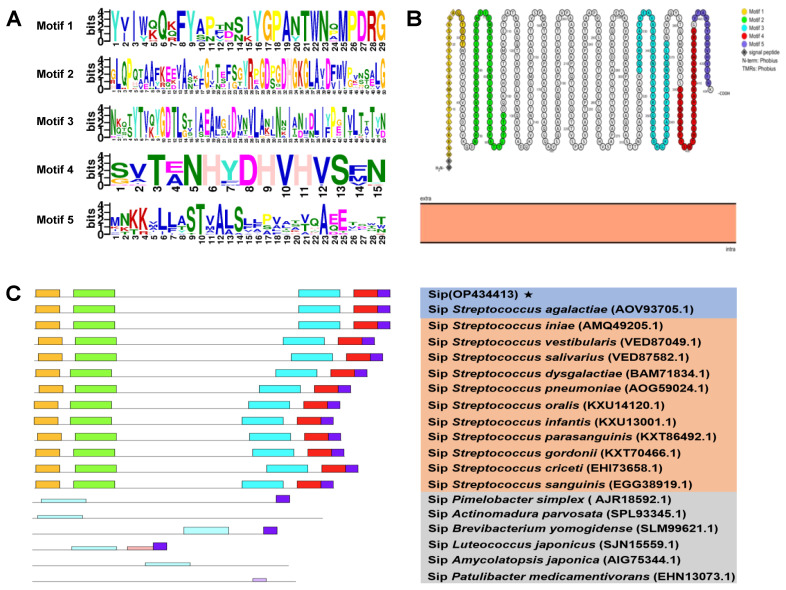
**Conserved motifs analysis of Sip with other 18 reference Sip.** (**A**) The symbols of top five conserved motifs with length ranging from 5 to 55 in Sip amino acid sequences. (**B**) The two-dimensional topology structure of Sip. (**C**) The location of these five motifs in bacteria Sip.

The accession number of the obtained nucleotide sequence in NCBI was OP434413. The result of CDD analysis indicated that there were three conserved domains in Sip amino acid sequence, i.e., the Lysin Motif (52–95 aa), the Treacle superfamily domain (110–314 aa) and the rne superfamily domain (163–323 aa) (Figure 1). As shown in Figure 2, there were five conserved motifs in bacteria Sip proteins with lengths ranging from 5 to 50 (Figure 2A) and their respective positions in bacteria Sip proteins (Figure 2C). As shown in Figure 2C, all of the reference bacteria Sip possessed all five conserved motifs, which account for 36.8% of Sip length, suggesting that Sip from different species has been moderately conserved. Moreover, the two-dimensional topology structures of Sip depicted that Sip was located extracellularly, and the signal peptide sequences were within the Motif 1 domain (Figure 2B).

**Figure 3:**
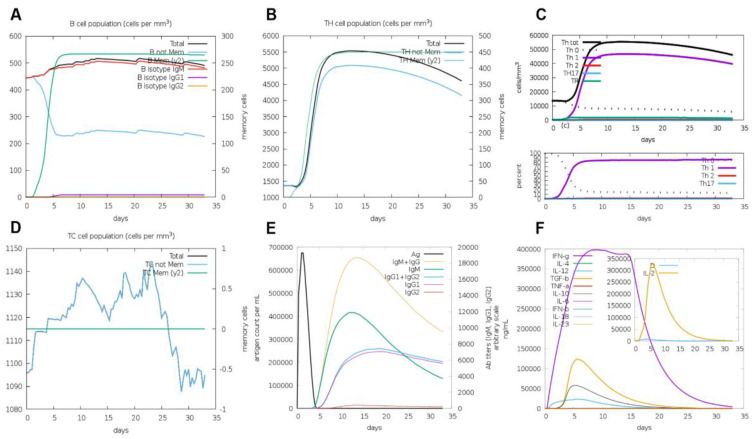
**Simulated immune stimulation by C-ImmSim software.** (**A**) Immune B cells population, (**B**) Th cells population; (**C**) the subtypes of Th cells; (**D**) TC cells population; (**E**) trends of immunoglobulin; (**F**) cytokine and interleukin.

The results of C-ImmSim analysis showed that B cells and Th cells maintained high levels all the time (Figure 3A,B), while TC cells maintained high levels first and then decreased (Figure 3D). After immunization, the number and proportion of Th1 cells in Th cells increased significantly (Figure 3C). The result of antibody secretion after immunization showed a trend of increasing first and then decreasing (Figure 3E). The changes in immune factors showed that the levels of IFN g and IL-2 increased significantly (Figure 3F).

### 3.2. Protein Expression and Immunogenicity Analysis of Sip

**Figure 4:**
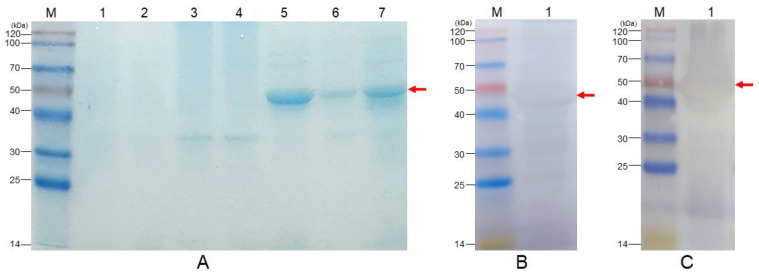
**SDS-PAGE and Western blotting analysis of rSip**. (**A**) SDS-PAGE analysis of rSip. M: Protein Marker (14-120 kDa), Lane 1: Uninduced BL21 (pET28a), Lane 2: Induced BL21 (pET28a), Lane 3: Uninduced BL21 (pET28a-Sip) supernatant, Lane 4: Uninduced BL21 (pET28a-Sip) precipitation, Lane 5: Induced BL21 (pET28a-Sip) supernatant, Lane 6: Induced BL21 (pET28a-Sip) precipitation, Lane 7: BNC-rSip. (**B**) The specific reaction of rSip with rabbit anti-*S. agalactiae*. (**C**) Specific reaction of rSip with rabbit anti-*S. iniae*. M: Protein Marker (14–120 kDa).

SDS-PAGE analysis showed that rSip could be expressed not only in the inclusion body but also in soluble form, and the BNC-rSip sample contained the target protein rSip (Figure 4A). The results of Western blotting indicated that rSip could specifically bind to rabbit anti-*S. agalactiae* (Figure 4B) and anti-*S. iniae* (Figure 4C), respectively, and showed the expected size bands.

### 3.3. Serum Specific Antibody Levels

**Figure 5:**
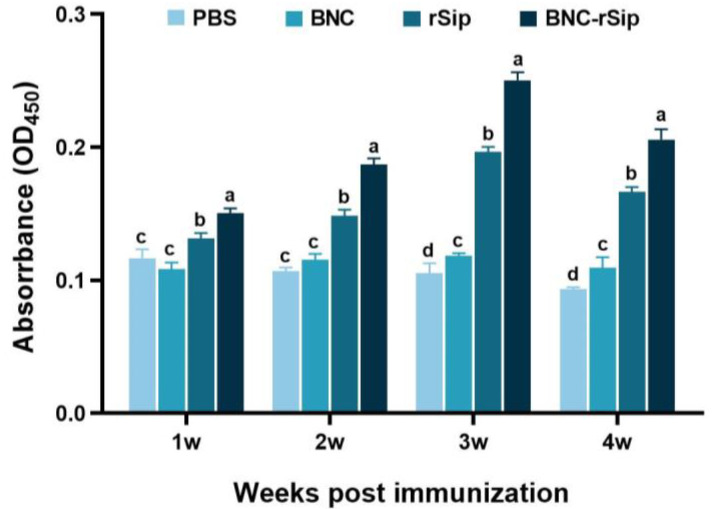
**Detection of serum antibody levels by ELISA.** Different letters (a, b, c, and d) suggested significant differences (*p* < 0.05, *n* = 4).

Immunized tilapia serum was collected at 1 wpi, 2 wpi, 3 wpi, and 4 wpi, and serum-specific antibody was determined using ELISA. According to the results, the serum antibody levels of the PBS control group and the BNC vector group had no significant changes from 1 to 4 wpi. The serum antibody levels were highest at 3 wpi in both the rSip group and the BNC-rSip group and were significantly (*p* < 0.05) higher than those of PBS and BNC groups from 1 to 4 wpi. Moreover, the serum antibody levels in the BNC-rSip group were significantly (*p* < 0.05) higher than those of the rSip group from 1 to 4 wpi (Figure 5).

### 3.4. Serum Enzyme Activity

**Figure 6:**
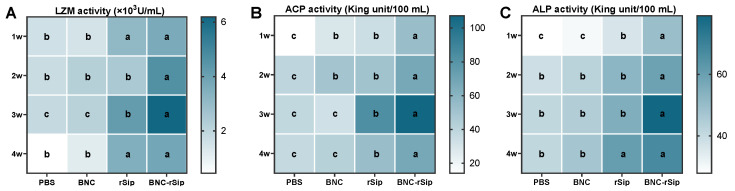
**Identification of serum enzyme activities at 1–4 wpi.** (**A**) LZM activity. (**B**) ACP activity. (**C**) ALP activity. Different letters (a, b, and c) indicated significant differences (*p* < 0.05, *n* = 4).

The determination of serum LZM, ACP, and ALP activities from 1 to 4 wpi were conducted. Compared with PBS and BNC groups, rSip and BNC-rSip groups showed significantly higher serum enzyme activity (*p* < 0.05) during the experiment. Additionally, the activities of four enzymes in rSip and BNC-rSip groups all peaked at 3 wpi, and the highest activities of BNC-rSip group were all significantly (*p* < 0.05) higher than those of the rSip group (Figure 6).

### 3.5. Immune-Related Genes Expression

**Figure 7:**
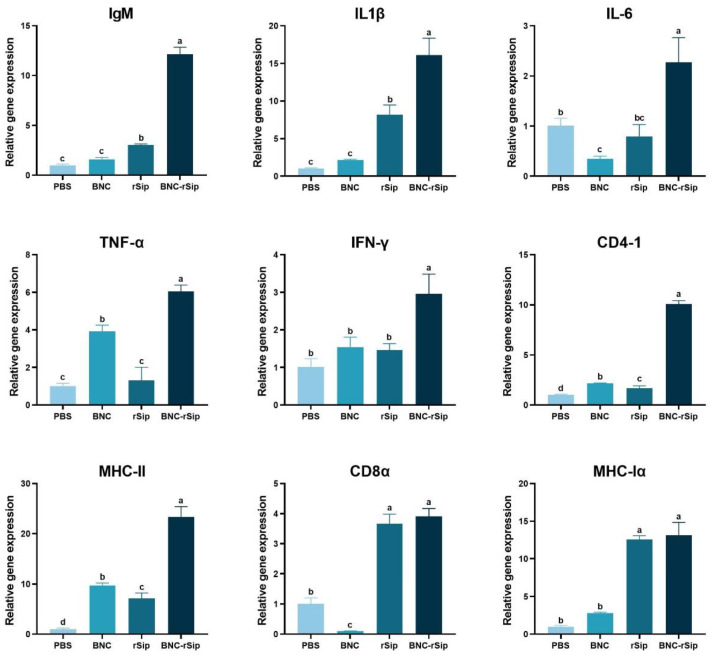
**The expression levels of immune-related genes in the spleen at 3 wpi.** Different letters (a, b, c, and d) suggested significant differences (*p* < 0.05, *n* = 4).

Spleens were collected at 3 wpi and the expression levels of immune-related genes (IgM, IL1β, IL-6, TNF-α, IFN-γ, CD4-1, CD8α, MHC Iα, MHC II) were detected via RT-qPCR, with EF1α as the internal reference gene. Figure 7 showed that all genes expressed at the highest levels in the BNC-rSip group compared with other groups (PBS, BNC, and rSip groups). MHC II gene expression level was the highest in the BNC-rSip group with 23.1-fold of the PBS group, while IL-6 gene expression level was the lowest with 2.3-fold of the PBS group. The expression levels of all detected genes in rSip group were higher than those of the PBS group, except for IL-6 in the rSip group, while most of the detected genes were significantly (*p* < 0.05) lower compared with the BNC-rSip group (Figure 7).

### 3.6. Protective Efficacy of BNC-rSip

**Figure 8:**
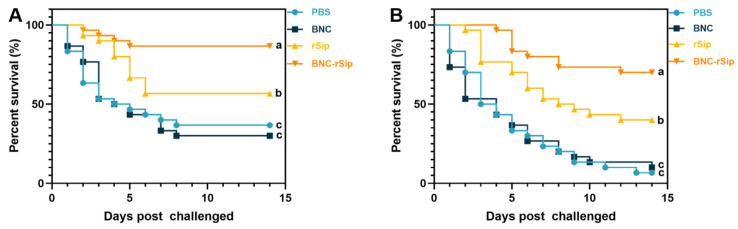
The percent survival during 14 days post challenge. (**A**) *S. agalactiae* challenge test, (**B**) *S. iniae* challenge test. Different letters (a, b, and c) suggested significant differences (*p* < 0.05).

After 4 weeks of immunization, tilapia was challenged with *S. agalactiae* and *S. iniae*, respectively, and took notes on the mortality for 14 days after the challenge. The results indicated that in the *S. agalactiae* challenge test, the survival rates were 36.67%, 30%, 56.67%, and 86.67% in PBS, BNC, rSip, and BNC-rSip groups (Figure 8A), respectively, and the corresponding RPS of the rSip and BNC-rSip group was 31.58% and 78.95%, respectively. In the *S. iniae* challenge test, the survival rates were 6.67%, 10%, 40%, and 70% in PBS, BNC, rSip, and BNC-rSip groups (Figure 8B), respectively, and the corresponding RPS of rSip and BNC-rSip group was 35.71% and 67.86%, respectively. Moreover, in the *S. agalactiae* and *S. iniae* challenge test, the survival rates of BNC-rSip group were both notably higher (*p* < 0.05) than those of rSip groups.

## 4. Discussion

Streptococcosis is a highly serious disease caused by *S. agalactiae* and *S. iniae* in freshwater fish farming, especially in the tilapia industry [21,22]. Compared with antibiotics and chemicals, vaccination is a green, safe, and ideal measure to prevent and control bacterial diseases in fish [23]. Among various vaccination methods, immersion immunization is convenient to operate, with low labor and costs, avoiding damage to fish body surface and stress response, but the immune effect of the immersion vaccine is not ideal because of the biological barrier of fish skin. BNCs have been proved to hold the potential as a carrier due to the following characteristics: excellent permeability, stiffness, low density, biocompatibility, and renewability, which will benefit the design of the advanced drug and protein delivery systems [14,15].

Many studies have shown that Sip is an ideal immunogenic protein which plays a crucial role in protecting from *Streptococcus* species infection [24]. In our previous research, there was a high similarity (99.8%) between the Sip amino acid sequences of *S. agalactiae* and *S. iniae* (data not shown), which suggested Sip was a conserved protein and may serve as the common antigen for the vaccines of *S. agalactiae* and *S. iniae*. Many studies have shown that C-ImmSim could model features of both the specific and innate immunity in fish [25,26]. In research by Madonia et al., the C-ImmSim software was used to simulate the immune response of sea bass by selecting the specific immunological and hematological features of fish [26]. In this study, C-ImmSim was used to simulate Sip immunity, and the results indicated that the vaccine encoding Sip could well induce humoral immunity and cellular immunity. The Western blotting indicated that rSip could specifically bind to rabbit anti-S. agalactiae and anti-S. iniae, respectively, suggesting that rSip could have a cross-protective effect against both *S. agalactiae* and *S. iniae* infections (Figure 5). Additionally, we connected the rSip with the functionalized BNCs to successfully construct the nanocarrier subunit vaccine BNC-rSip.

Specific antibody is an indispensable proxy of the immune response induced by vaccination and infection, which is comprehensively used as a protection proxy for fish vaccines [27]. There was no significant change in serum antibody levels in the PBS and BNC groups, as they did not contain antigen protein. Meanwhile, the antibody levels of the rSip group and the BNC-rSip group increased significantly from 1 to 3 wpi, and achieved the peak at 3 wpi. Inevitably, the serum antibody level in BNC-rSip group was significantly higher (*p* < 0.05) than that of rSip group (Figure 6) from 1 to 4 wpi, which was similar to the finding that the chitosan complex nanovaccine of tilapia lake virus induced a higher antibody level than the inactivated virus group in tilapia [28], suggesting that BNC had a positive effect on immune response.

The nonspecific immune response, as the first line of immune defense, plays an important role in resisting the pathogenic microorganisms entering fish. Fish serum contains nonspecific immune enzymes, such as LZM and ACP, which play an indispensable role in nonspecific immunity. LZM is involved in a range of immune responses and plays a critical role in the resistance to foreign pathogenic bacteria, fungi, viruses, or tumor invasion. ACP is a kind of lysosomal enzyme extensively found in animals and plants. Abnormal ACP levels are often closely associated with various diseases of the individual [29]. In this study, the serum enzyme activities were significantly higher in the rSip group and the BNC-rSip group compared with PBS and BNC groups. Additionally, the activities of three enzymes in rSip and BNC-rSip groups all peaked at 3 wpi, and the highest activities of the BNC-rSip group were all higher than those of the rSip group (Figure 7), which demonstrated that BNC-rSip can dramatically improve the innate immunity level of tilapia.

In addition, our study investigated the expression of some immune-related genes to comprehensively analyze the immune effects of vaccines. IL-6 is a soluble mediator with a pleiotropic effect on hematopoiesis, immune response, and inflammation [30]. MHC-I and MHC-II molecules are of great importance in the immune system and autoimmunity [31], which have been reported to be present in teleost [32]. In response to pathogens, MHC-I molecule delivers endogenous antigen to Cytotoxic T cell through surface CD8 molecule, thereby releasing apoptosis-inducing molecules to directly kill infected cells [33,34], while the MHC-II molecule delivers exogenous antigen to a helper T cell through the surface CD4 molecule, discharging cytokines to kill pathogens indirectly. IFN-γ can induce or upregulate the expression of MHC-II molecules on the cell surface [35]. TNF-α plays several therapeutic roles in the body, which include immunostimulation, resistance to infectious agents, and resistance to tumors [36]. IL-1β is a potent proinflammatory cytokine which plays a crucial role in the regulation of inflammation and can induce the host defense mechanism during pathogen infection. In this study, compared with the PBS group, the expression levels of all detected genes in BNC-rSip group were upgraded with a variety of degrees (Figure 7); of these, the MHC II expression in BNC-rSip group was highest compared with other genes and other groups (Figure 7). In teleost fish, IgM is the most abundant immunoglobulin isotype [37] and is a major mediator of humoral immune response [38]. Our study found that the IgM expression level was also increased to some extent, which illustrated that an effective humoral immune response was induced, which was in line with the result of the immune-related genes expression of oral vaccinated tilapia against *S. agalactiae* infection [39].

Moreover, in the challenge test, the RPS of BNC-rSip groups were 78.95% (*S. agalactiae*) and 67.86% (*S. iniae*), respectively, and both much higher than that of other groups (Figure 8), which was consistent with the study’s findings that a novel chimeric multiepitope vaccine provided higher immuneprotection against streptococcosis disease in tilapia [40]. The above results verified that the nanocarrier subunit vaccine BNC-rSip can be a promising preventative measure against streptococcus infection in tilapia.

## 5. Conclusions

In this study, the nanocarrier vaccine system BNC-rSip was successfully constructed by linking the rSip and functionalized BNCs. Compared with PBS and BNC groups, BNC-rSip could effectively promote the serum antibody level, related enzyme activities, and immune-related genes expression. BNC-rSip induced a better immune effect, and the RPS were 78.95% (*S. agalactiae*) and 67.86% (*S. iniae*), much higher than those of rSip groups, respectively. In conclusion, the nanovaccine BNC-rSip induces a strong immune response via immersion immunization and has the potential to provide cross-protection against *S. agalactiae* and *S. iniae* infections in tilapia.

## Figures and Tables

**Table 1:** The groups of immersion vaccination.

Groups	Concentration	Fish No.	Immersion Time
PBS	—	70	8 h
BNC	10 mg/L	70	8 h
rSip	10 mg/L	70	8 h
BNC-rSip	10 mg/L *	70	8 h

Note: “*” refers to the effective concentration of rSip in BNC-rSip vaccine.

**Table 2:** The primers information used in the study.

Gene Name	Accession No.	Primer Sequences (from 5′ to 3′)
*EF1α*	AB075952.1	F: TGATCTACAAGTGCGGAGGAA R: GGAGCCCTTTCCCATCTCA
*IL-1β*	XM_019365841.2	F: AGCTCCATGCAGTGATGCTG R: TGTTTTTATCCGTCACCTCCTCC
*IL-6*	XM_019350387.2	F: GTGTGGCAGGTGACTTCTCA R: GGAAATGGTGCTCAAACGCT
*IFN-γ*	NM_001287402.1	F: GGAGACCCTCAGAGATATCAAGATGAATG R: GCGACCTTTAGCCTTTGTTTGCCT
*TNF-α*	NM_001279533	F: GCCATCCATCTAGAAGGCAGCGA R: GAAGTACAGGCCAGTGTGTGGGAT
*CD4-1*	XM_005455488.4	F: CCAAGGGAAACAGAGAAGGAAA R: AAGGGATGGTGAGAGGTGAAAC
*CD8α*	XM_005450353.4	F: CATAACAGCAAAGGAAGGACAG R: TACCTTGGATAAGTGACGCA
*IgM*	KF305823.1	F: CATCACCTATGACGAATGGACCAGGG R: GCGCTGAGGGTTTCCTCCAAAATC
*MHC I*	FJ457118.1	F: GATGAGGACAGATGTTCCCAAAGTGT R: CTCTCCTTTGTGCACACCATCATGA
*MHC II*	NM_001279562.1	F: ATCACTGACTGGGATCCCTCCTC R: CTCGAGCCTTCCTCCTGTAGTAGAT

## Data Availability

The data in this study are available from the corresponding author on reasonable request.
